# Supplementary material for: Water masses shape pico-nano eukaryotic communities of the Weddell Sea
Source: Commun Biol. 2023 Jan 18;6:64. doi: 10.1038/s42003-023-04452-7 (PMC9849203; doi:10.1038/s42003-023-04452-7)
Supplement: Supplementary file 2 — Supplementary Information [file 42003_2023_4452_MOESM2_ESM.pdf]

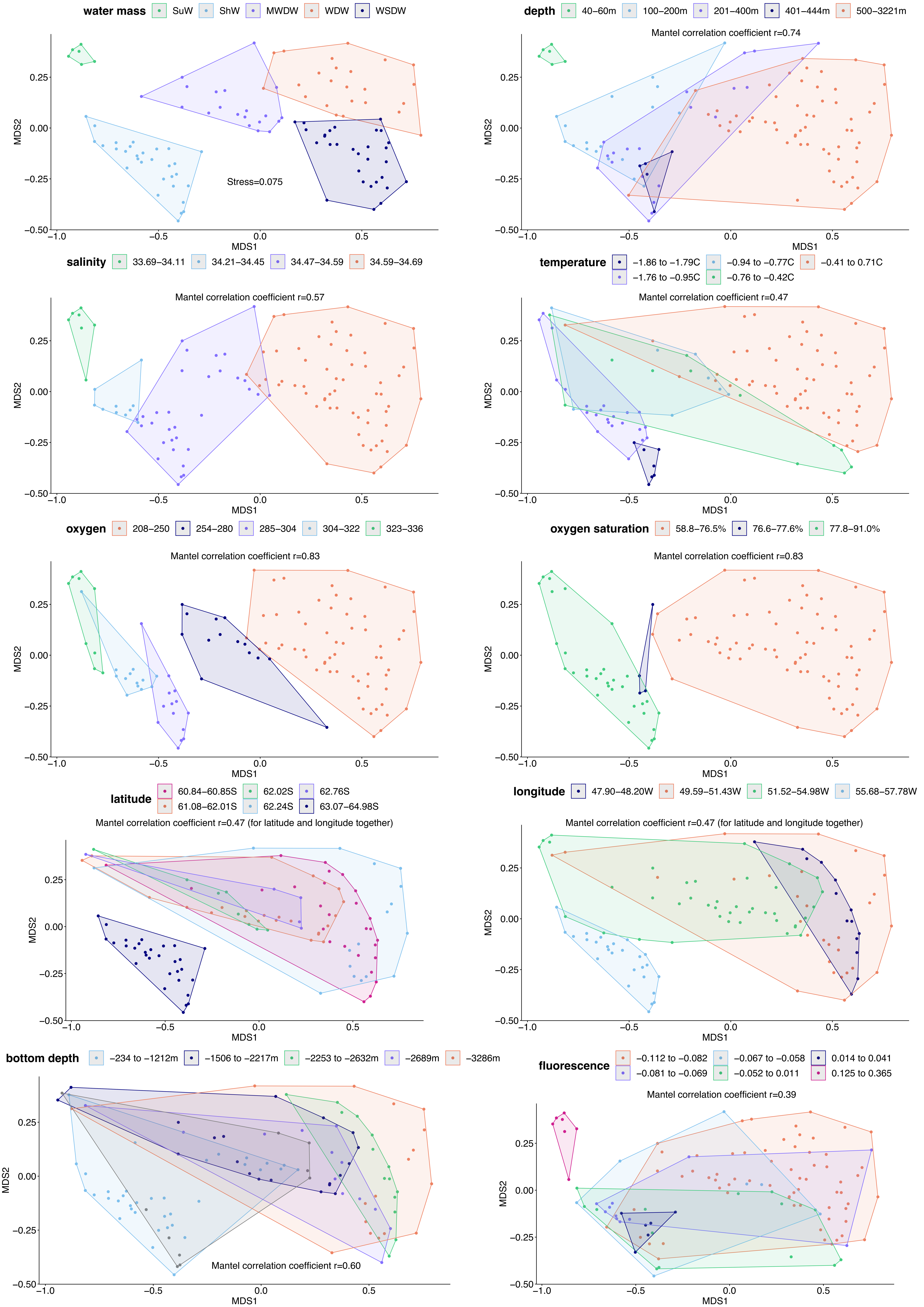

water mass
SuW
ShW
MWDW
WDW
WSDW
Stress=0.075
depth
40–60m
100–200m
201–400m
401–444m
500–3221m
Mantel correlation coefficient r=0.74
salinity
33.69–34.11
34.21–34.45
34.47–34.59
34.59–34.69
Mantel correlation coefficient r=0.57
temperature
−1.86 to −1.79C
−0.94 to −0.77C
−0.41 to 0.71C
−1.76 to −0.95C
−0.76 to −0.42C
Mantel correlation coefficient r=0.47
oxygen
208–250
254–280
285–304
304–322
323–336
Mantel correlation coefficient r=0.83
oxygen saturation
58.8–76.5%
76.6–77.6%
77.8–91.0%
Mantel correlation coefficient r=0.83
latitude
60.84–60.85S
62.02S
62.76S
61.08–62.01S
62.24S
63.07–64.98S
Mantel correlation coefficient r=0.47 (for latitude and longitude together)
longitude
47.90–48.20W
49.59–51.43W
51.52–54.98W
55.68–57.78W
Mantel correlation coefficient r=0.47 (for latitude and longitude together)
bottom depth
−234 to −1212m
−1506 to −2217m
−2253 to −2632m
−2689m
−3286m
Mantel correlation coefficient r=0.60
fluorescence
−0.112 to −0.082
−0.067 to −0.058
0.014 to 0.041
−0.081 to −0.069
−0.052 to 0.011
0.125 to 0.365
Mantel correlation coefficient r=0.39
MDS1
MDS2

Suppl. Fig. 1. Two-dimensional NMDS plots illustrating pairwise Bray-Curtis distances between pico-nano eukaryotic communities in the Western Weddell Sea. All NMDS plots are identical (with a stress value = 0.075 is shown in panel **A**), but the samples are clustered in different ways as described below. The water masses are mapped on the NMDS plot in the upper left corner, along with the environmental variables measured in this study and geographical coordinates. Boundary points on gradients of those variables (where pico-nano eukaryotic community composition is significantly different across the boundary) were identified using a split moving-window analysis of ecological differentiation based on a Z-score cutoff of 1.2 , and samples were clustered according to these boundary points. The clusters are colored and marked with polygons. The intervals of the variable corresponding to the clusters are shown in the legends on top of each plot. Mantel correlation coefficients (calculated for a matrix of Bray-Curtis distances between the pico-nano eukaryotic communities and for a matrix of Bray-Curtis distances between the samples based on a variable) are shown for each continuous variable.

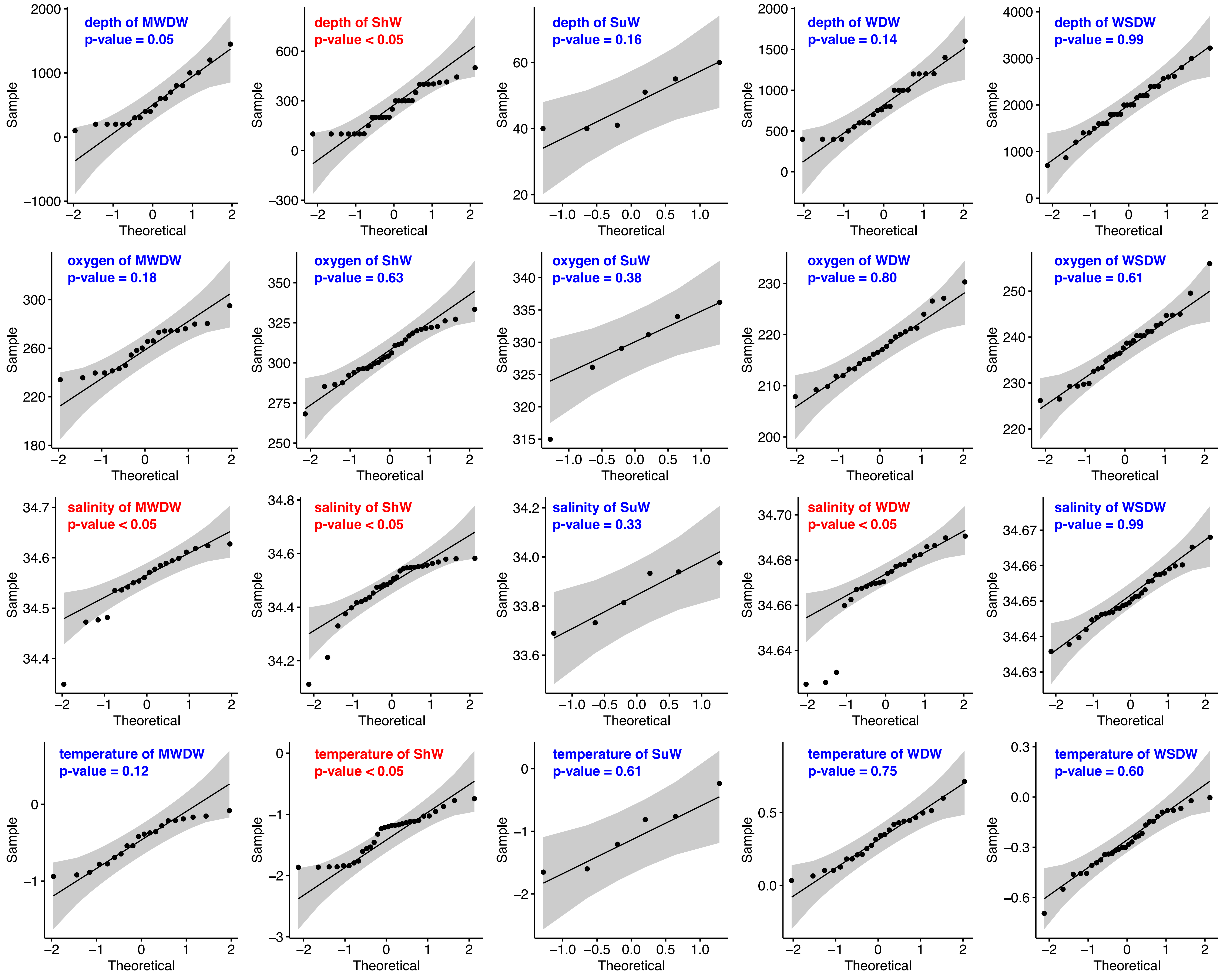
depth of MWDW
p-value = 0.05
depth of ShW
p-value < 0.05
depth of SuW
p-value = 0.16
depth of WDW
p-value = 0.14
depth of WSDW
p-value = 0.99
oxygen of MWDW
p-value = 0.18
oxygen of ShW
p-value = 0.63
oxygen of SuW
p-value = 0.38
oxygen of WDW
p-value = 0.80
oxygen of WSDW
p-value = 0.61
salinity of MWDW
p-value < 0.05
salinity of ShW
p-value < 0.05
salinity of SuW
p-value = 0.33
salinity of WDW
p-value < 0.05
salinity of WSDW
p-value = 0.99
temperature of MWDW
p-value = 0.12
temperature of ShW
p-value < 0.05
temperature of SuW
p-value = 0.61
temperature of WDW
p-value = 0.75
temperature of WSDW
p-value = 0.60
Sample
Theoretical

Suppl. Fig. 2. Normality test results for depth, oxygen concentration, salinity, and temperature across five water masses. Quantile-Quantile plots, confidence intervals and p-values of the Shapiro-Wilk test are shown.

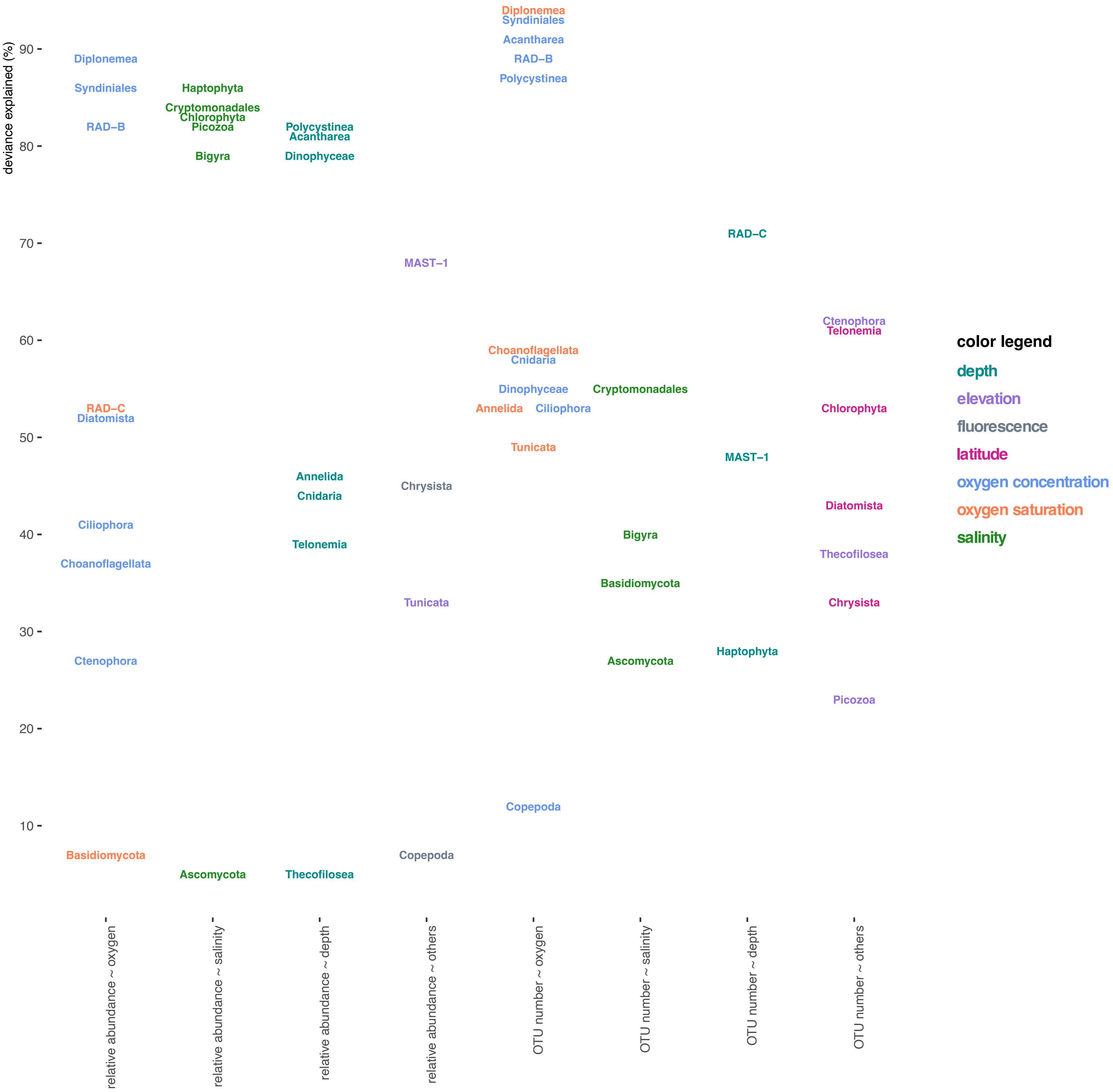

deviance explained (%)
90
80
70
60
50
40
30
20
10
Diplonemea
Syndiniales
RAD−B
RAD−C
Diatomista
Ciliophora
Choanoflagellata
Ctenophora
Basidiomycota
Haptophyta
Cryptomonadales
Chlorophyta
Picozoa
Bigyra
Ascomycota
Polycystinea
Acantharea
Dinophyceae
Annelida
Cnidaria
Telonemia
Thecofilosea
MAST−1
Chrysista
Tunicata
Copepoda
Diplonemea
Syndiniales
Acantharea
RAD−B
Polycystinea
Choanoflagellata
Cnidaria
Dinophyceae
Annelida
Ciliophora
Tunicata
Copepoda
Cryptomonadales
Bigyra
Basidiomycota
Ascomycota
RAD−C
MAST−1
Haptophyta
Ctenophora
Telonemia
Chlorophyta
Diatomista
Thecofilosea
Chrysista
Picozoa
relative abundance ~ oxygen
relative abundance ~ salinity
relative abundance ~ depth
relative abundance ~ others
OTU number ~ oxygen
OTU number ~ salinity
OTU number ~ depth
OTU number ~ others
color legend
depth
elevation
fluorescence
latitude
oxygen concentration
oxygen saturation
salinity

Suppl. Fig. 3. A summary of GAM results showing influence of abiotic variables on relative abundance or OTU richness of 26 eukaryotic clades. Percentages of variance explained by GAM models are shown for clade/variable pairs on the y-axis. The environmental variables are coded by color and grouped into four categories: depth, oxygen, salinity, and the other variables. For relative abundance or OTU richness of each clade, only the highest variance explained across all the environmental variables is shown, hence each clade appears in one category only.

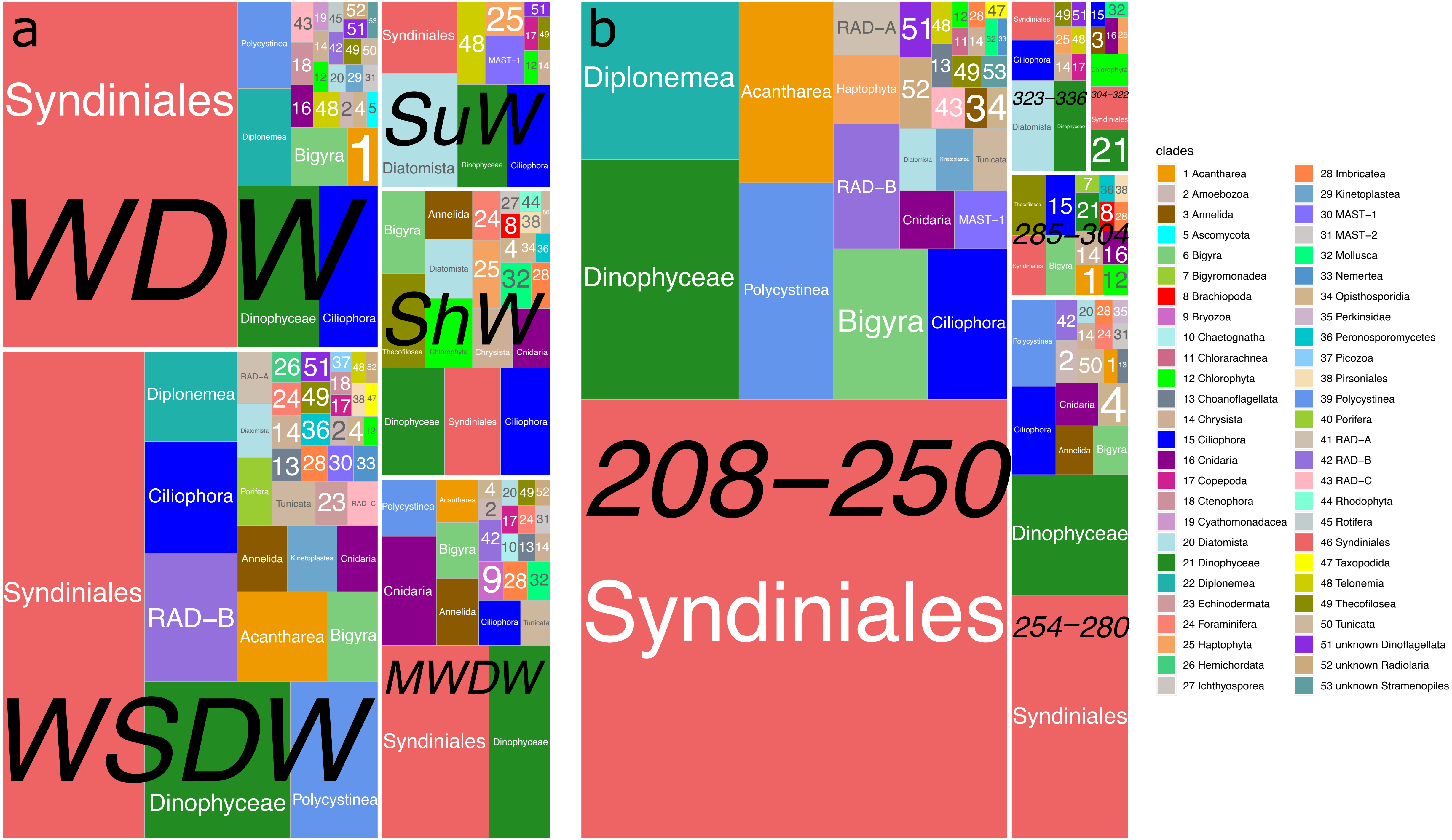
a
WDW
SuW
ShW
WSDW
MWDW
b
208–250
323–336
304–322
285–304
254–280
clades
1 Acantharea
2 Amoebozoa
3 Annelida
5 Ascomycota
6 Bigyra
7 Bigyromonadea
8 Brachiopoda
9 Bryozoa
10 Chaetognatha
11 Chlorarachnea
12 Chlorophyta
13 Choanoflagellata
14 Chrysista
15 Ciliophora
16 Cnidaria
17 Copepoda
18 Ctenophora
19 Cyathomonadacea
20 Diatomista
21 Dinophyceae
22 Diplonemea
23 Echinodermata
24 Foraminifera
25 Haptophyta
26 Hemichordata
27 Ichthyosporea
28 Imbricatea
29 Kinetoplastea
30 MAST–1
31 MAST–2
32 Mollusca
33 Nemertea
34 Opisthosporidia
35 Perkinsidae
36 Peronosporomycetes
37 Picozoa
38 Pirsoniales
39 Polycystinea
40 Porifera
41 RAD–A
42 RAD–B
43 RAD–C
44 Rhodophyta
45 Rotifera
46 Syndiniales
47 Taxopodida
48 Telonemia
49 Thecofilosea
50 Tunicata
51 unknown Dinoflagellata
52 unknown Radiolaria
53 unknown Stramenopiles

Suppl. Fig. 4. Square pie charts illustrating taxonomy of OTUs that were identified as "indicators" for one of five water masses (panel **a**) or one of five intervals of oxygen concentration (panel **b**) separated by the ecological boundaries (Fig. 2). Taxonomic groups are labelled and numbered according to the legend. The water masses or oxygen concentration intervals are labelled in black.

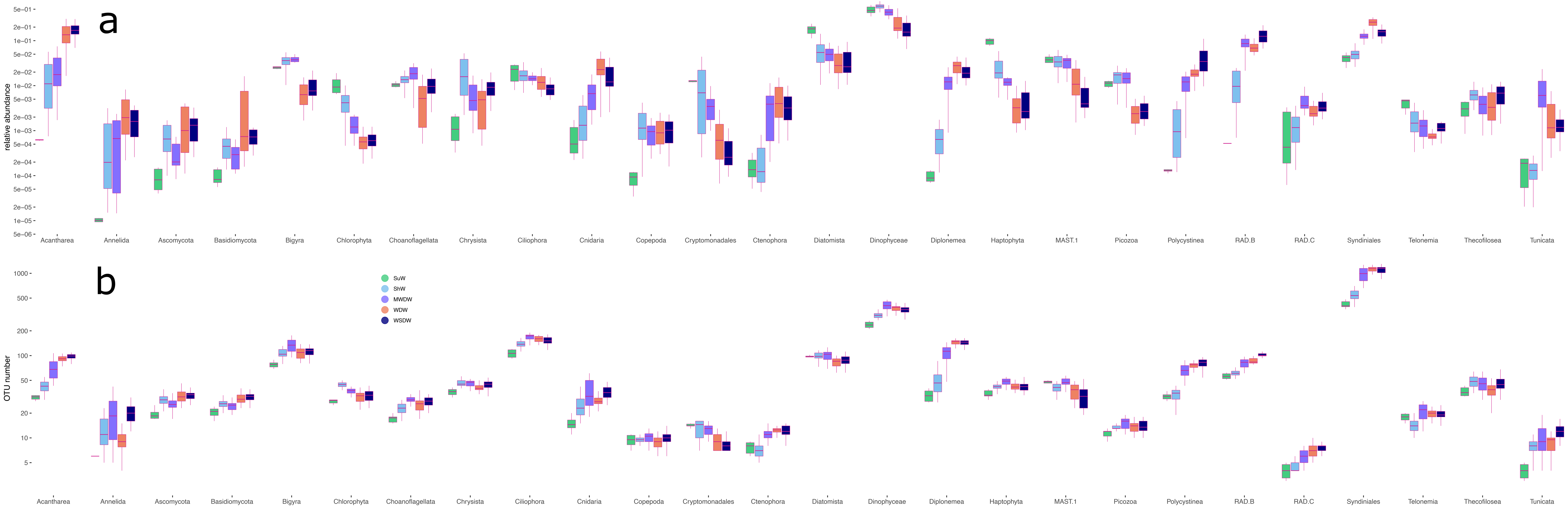
a
relative abundance
5e−01
2e−01
1e−01
5e−02
2e−02
1e−02
5e−03
2e−03
1e−03
5e−04
2e−04
1e−04
5e−05
2e−05
1e−05
5e−06
Acantharea
Annelida
Ascomycota
Basidiomycota
Bigyra
Chlorophyta
Choanoflagellata
Chrysista
Ciliophora
Cnidaria
Copepoda
Cryptomonadales
Ctenophora
Diatomista
Dinophyceae
Diplonemea
Haptophyta
MAST.1
Picozoa
Polycystinea
RAD.B
RAD.C
Syndiniales
Telonemia
Thecofilosea
Tunicata
b
OTU number
1000
500
200
100
50
20
10
5
SuW
ShW
MWDW
WDW
WSDW
Acantharea
Annelida
Ascomycota
Basidiomycota
Bigyra
Chlorophyta
Choanoflagellata
Chrysista
Ciliophora
Cnidaria
Copepoda
Cryptomonadales
Ctenophora
Diatomista
Dinophyceae
Diplonemea
Haptophyta
MAST.1
Picozoa
Polycystinea
RAD.B
RAD.C
Syndiniales
Telonemia
Thecofilosea
Tunicata

Suppl. Fig. 5. Distribution of 26 eukaryotic taxonomic groups across the five water masses. Relative abundance (**a**) or OTU richness (**b**) values are shown in the form of boxplots. The y-axis scales are logarithmic.

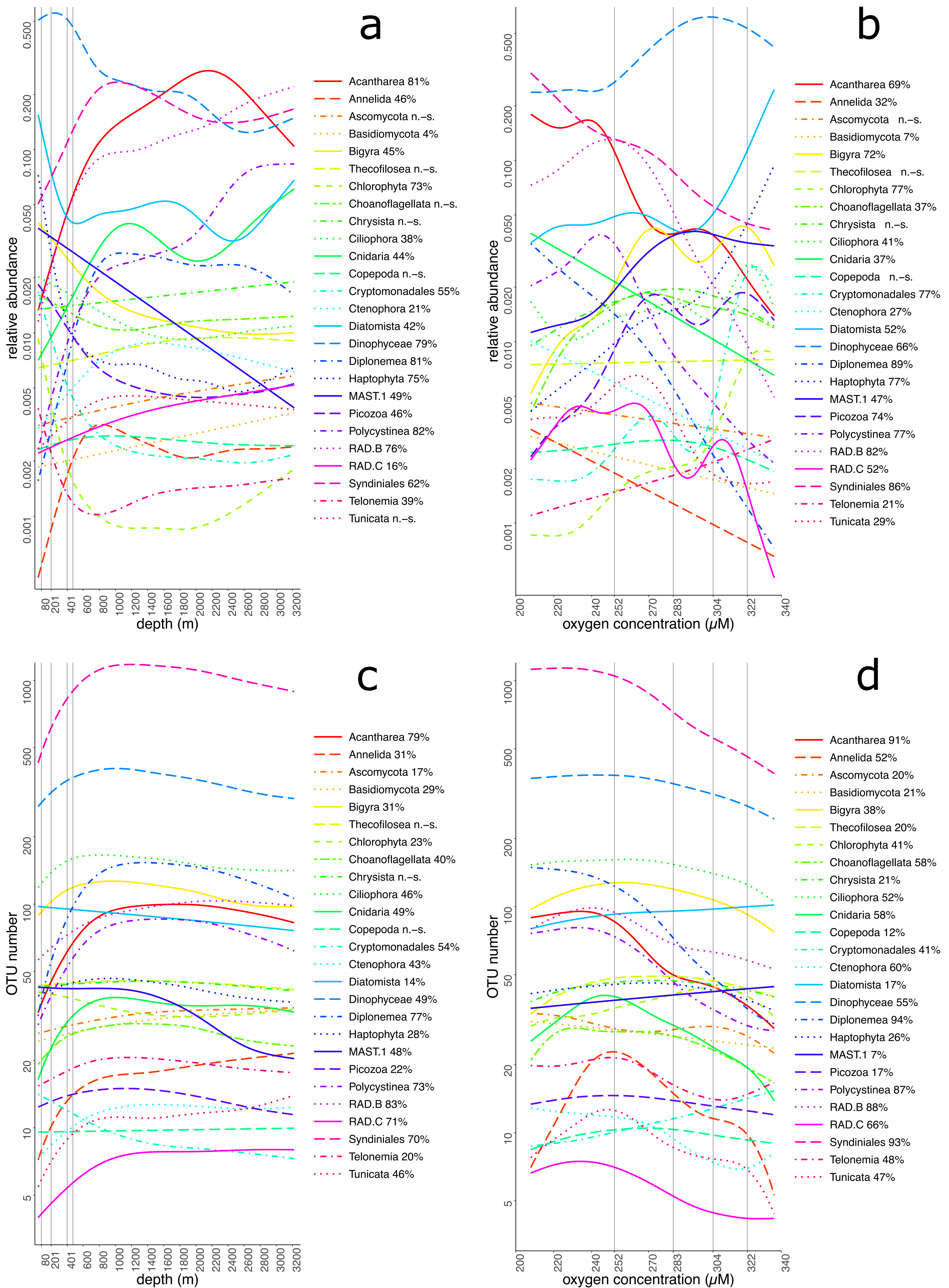

a
relative abundance
depth (m)
Acantharea 81%
Annelida 46%
Ascomycota n.–s.
Basidiomycota 4%
Bigyra 45%
Thecofilosea n.–s.
Chlorophyta 73%
Choanoflagellata n.–s.
Chrysista n.–s.
Ciliophora 38%
Cnidaria 44%
Copepoda n.–s.
Cryptomonadales 55%
Ctenophora 21%
Diatomista 42%
Dinophyceae 79%
Diplonemea 81%
Haptophyta 75%
MAST.1 49%
Picozoa 46%
Polycystinea 82%
RAD.B 76%
RAD.C 16%
Syndiniales 62%
Telonemia 39%
Tunicata n.–s.
b
relative abundance
oxygen concentration (μM)
Acantharea 69%
Annelida 32%
Ascomycota n.–s.
Basidiomycota 7%
Bigyra 72%
Thecofilosea n.–s.
Chlorophyta 77%
Choanoflagellata 37%
Chrysista n.–s.
Ciliophora 41%
Cnidaria 37%
Copepoda n.–s.
Cryptomonadales 77%
Ctenophora 27%
Diatomista 52%
Dinophyceae 66%
Diplonemea 89%
Haptophyta 77%
MAST.1 47%
Picozoa 74%
Polycystinea 77%
RAD.B 82%
RAD.C 52%
Syndiniales 86%
Telonemia 21%
Tunicata 29%
c
OTU number
depth (m)
Acantharea 79%
Annelida 31%
Ascomycota 17%
Basidiomycota 29%
Bigyra 31%
Thecofilosea n.–s.
Chlorophyta 23%
Choanoflagellata 40%
Chrysista n.–s.
Ciliophora 46%
Cnidaria 49%
Copepoda n.–s.
Cryptomonadales 54%
Ctenophora 43%
Diatomista 14%
Dinophyceae 49%
Diplonemea 77%
Haptophyta 28%
MAST.1 48%
Picozoa 22%
Polycystinea 73%
RAD.B 83%
RAD.C 71%
Syndiniales 70%
Telonemia 20%
Tunicata 46%
d
OTU number
oxygen concentration (μM)
Acantharea 91%
Annelida 52%
Ascomycota 20%
Basidiomycota 21%
Bigyra 38%
Thecofilosea 20%
Chlorophyta 41%
Choanoflagellata 58%
Chrysista 21%
Ciliophora 52%
Cnidaria 58%
Copepoda 12%
Cryptomonadales 41%
Ctenophora 60%
Diatomista 17%
Dinophyceae 55%
Diplonemea 94%
Haptophyta 26%
MAST.1 7%
Picozoa 17%
Polycystinea 87%
RAD.B 88%
RAD.C 66%
Syndiniales 93%
Telonemia 48%
Tunicata 47%

Suppl. Fig. 6. Plots illustrating relative abundance (**a** , **b**) or OTU richness (**c** , **d**) of 26 eukaryotic taxonomic groups changing with depth (**a** , **c**) or oxygen concentration (**b**, **d**) in the Western Weddell Sea. Only trend lines calculated using the GAM approach are shown along with percentage of variance explained. Confidence intervals for GAMs and individual data points are not shown for simplicity. The y-axes scales are logarithmic. Oxygen concentration or depth values identified as ecological boundary points are marked with vertical grey lines.

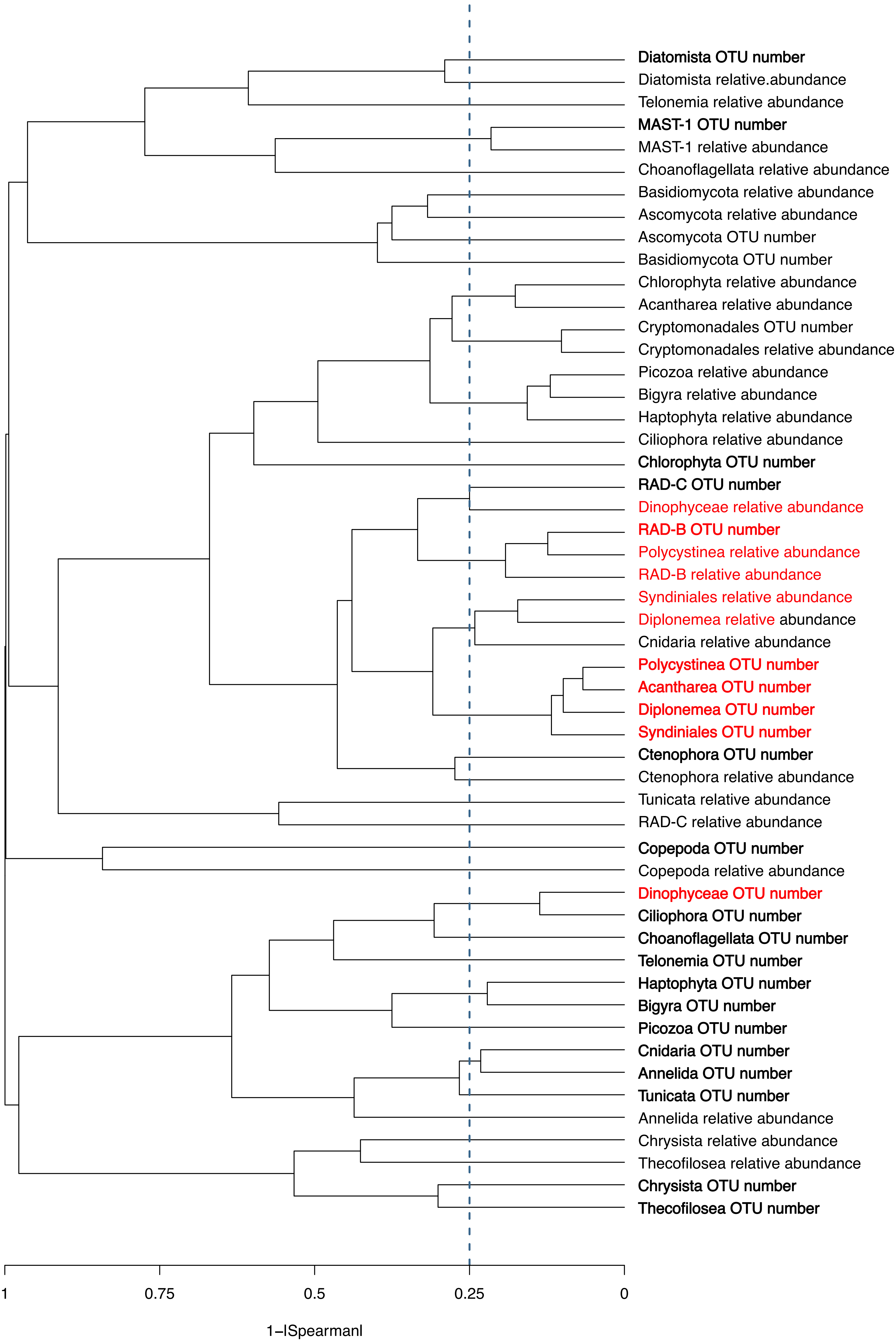
Diatomista OTU number
Diatomista relative.abundance
Telonemia relative abundance
MAST-1 OTU number
MAST-1 relative abundance
Choanoflagellata relative abundance
Basidiomycota relative abundance
Ascomycota relative abundance
Ascomycota OTU number
Basidiomycota OTU number
Chlorophyta relative abundance
Acantharea relative abundance
Cryptomonadales OTU number
Cryptomonadales relative abundance
Picozoa relative abundance
Bigyra relative abundance
Haptophyta relative abundance
Ciliophora relative abundance
Chlorophyta OTU number
RAD-C OTU number
Dinophyceae relative abundance
RAD-B OTU number
Polycystinea relative abundance
RAD-B relative abundance
Syndiniales relative abundance
Diplonemea relative abundance
Cnidaria relative abundance
Polycystinea OTU number
Acantharea OTU number
Diplonemea OTU number
Syndiniales OTU number
Ctenophora OTU number
Ctenophora relative abundance
Tunicata relative abundance
RAD-C relative abundance
Copepoda OTU number
Copepoda relative abundance
Dinophyceae OTU number
Ciliophora OTU number
Choanoflagellata OTU number
Telonemia OTU number
Haptophyta OTU number
Bigyra OTU number
Picozoa OTU number
Cnidaria OTU number
Annelida OTU number
Tunicata OTU number
Annelida relative abundance
Chrysista relative abundance
Thecofilosea relative abundance
Chrysista OTU number
Thecofilosea OTU number
1
0.75
0.5
0.25
0
1−|Spearman|

Suppl. Fig. 7. A dendrogram visualizing a matrix of pairwise Spearman correlation coefficients (1 – |Spearman coefficient|) for relative abundance and OTU richness of 26 eukaryotic clades. Most important members of the deep-sea community are highlighted in red.

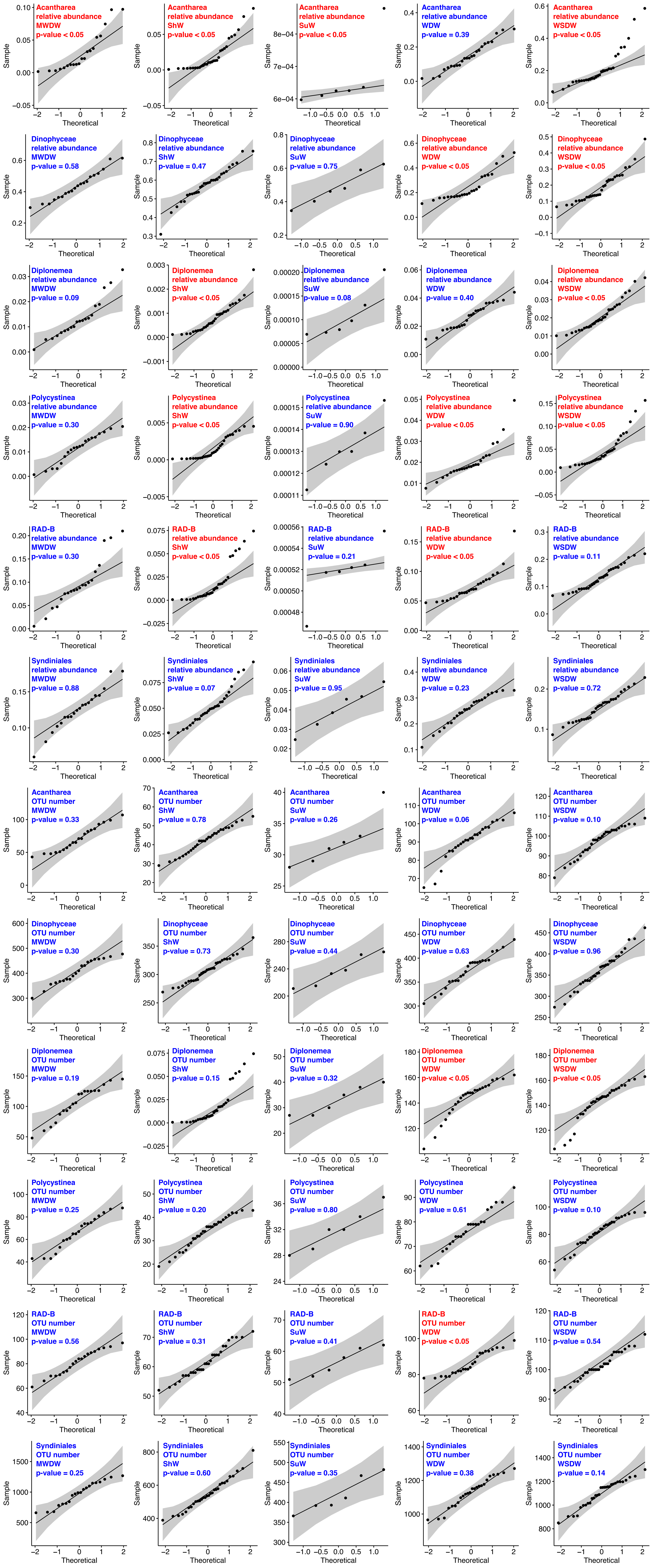

Acantharea relative abundance MWDW p-value < 0.05
Acantharea relative abundance ShW p-value < 0.05
Acantharea relative abundance SuW p-value < 0.05
Acantharea relative abundance WDW p-value = 0.39
Acantharea relative abundance WSDW p-value < 0.05
Dinophyceae relative abundance MWDW p-value = 0.58
Dinophyceae relative abundance ShW p-value = 0.47
Dinophyceae relative abundance SuW p-value = 0.75
Dinophyceae relative abundance WDW p-value < 0.05
Dinophyceae relative abundance WSDW p-value < 0.05
Diplonemea relative abundance MWDW p-value = 0.09
Diplonemea relative abundance ShW p-value < 0.05
Diplonemea relative abundance SuW p-value = 0.08
Diplonemea relative abundance WDW p-value = 0.40
Diplonemea relative abundance WSDW p-value < 0.05
Polycystinea relative abundance MWDW p-value = 0.30
Polycystinea relative abundance ShW p-value < 0.05
Polycystinea relative abundance SuW p-value = 0.90
Polycystinea relative abundance WDW p-value < 0.05
Polycystinea relative abundance WSDW p-value < 0.05
RAD-B relative abundance MWDW p-value = 0.30
RAD-B relative abundance ShW p-value < 0.05
RAD-B relative abundance SuW p-value = 0.21
RAD-B relative abundance WDW p-value < 0.05
RAD-B relative abundance WSDW p-value = 0.11
Syndiniales relative abundance MWDW p-value = 0.88
Syndiniales relative abundance ShW p-value = 0.07
Syndiniales relative abundance SuW p-value = 0.95
Syndiniales relative abundance WDW p-value = 0.23
Syndiniales relative abundance WSDW p-value = 0.72
Acantharea OTU number MWDW p-value = 0.33
Acantharea OTU number ShW p-value = 0.78
Acantharea OTU number SuW p-value = 0.26
Acantharea OTU number WDW p-value = 0.06
Acantharea OTU number WSDW p-value = 0.10
Dinophyceae OTU number MWDW p-value = 0.30
Dinophyceae OTU number ShW p-value = 0.73
Dinophyceae OTU number SuW p-value = 0.44
Dinophyceae OTU number WDW p-value = 0.63
Dinophyceae OTU number WSDW p-value = 0.96
Diplonemea OTU number MWDW p-value = 0.19
Diplonemea OTU number ShW p-value = 0.15
Diplonemea OTU number SuW p-value = 0.32
Diplonemea OTU number WDW p-value < 0.05
Diplonemea OTU number WSDW p-value < 0.05
Polycystinea OTU number MWDW p-value = 0.25
Polycystinea OTU number ShW p-value = 0.20
Polycystinea OTU number SuW p-value = 0.80
Polycystinea OTU number WDW p-value = 0.61
Polycystinea OTU number WSDW p-value = 0.10
RAD-B OTU number MWDW p-value = 0.56
RAD-B OTU number ShW p-value = 0.31
RAD-B OTU number SuW p-value = 0.41
RAD-B OTU number WDW p-value < 0.05
RAD-B OTU number WSDW p-value = 0.54
Syndiniales OTU number MWDW p-value = 0.25
Syndiniales OTU number ShW p-value = 0.60
Syndiniales OTU number SuW p-value = 0.35
Syndiniales OTU number WDW p-value = 0.38
Syndiniales OTU number WSDW p-value = 0.14
Sample
Theoretical

Suppl. Fig. 8. Normality test results for relative abundance values and OTU counts of six key players in the deep-sea eukaryotic community distributed across five water masses. Quantile-Quantile plots, confidence intervals and p-values of the Shapiro-Wilk test are shown.
