# Supplementary material for: Water masses shape pico-nano eukaryotic communities of the Weddell Sea
Source: Commun Biol. 2023 Jan 18;6:64. doi: 10.1038/s42003-023-04452-7 (PMC9849203; doi:10.1038/s42003-023-04452-7)
Supplement: Supplementary file 3 — Description of Additional Supplementary Files [file 42003_2023_4452_MOESM3_ESM.pdf]

Description of Additional Supplementary Files

File name: Supplementary Data 1

Description: A table containing main information about the samples and is the source data behind Figures 1, 2, and 4 in the paper

File name: Supplementary Data 2

Description: Relative abundance matrix of 5,048 OTUs, which is the source data behind the Figure 2 in the paper

File name: Supplementary Data 3

Description: Summary of taxonomical annotation of sampled OTUs

File name: Supplementary Data 4

Description: Results of non-linear correlations between continuous environmental variables and the relative abundance or OTU richness of sampled clades

File name: Supplementary Data 5

Description: The results of the analysis for the identification of indicator OTUs

File name: Supplementary Data 6

Description: The source data behind Figures 3a

File name: Supplementary Data 7

Description: The source data behind Figures3b
